# Efficacy of extracorporeal plasma therapy for adult native kidney patients with Primary FSGS: a Systematic review

**DOI:** 10.1080/0886022X.2023.2176694

**Published:** 2023-02-10

**Authors:** Jing Miao, Pajaree Krisanapan, Supawit Tangpanithandee, Charat Thongprayoon, Michael A. Mao, Wisit Cheungpasitporn

**Affiliations:** a Division of Nephrology and Hypertension, Department of Medicine, Mayo Clinic, Rochester, MN, USA; b Division of Nephrology, Department of Internal Medicine, Faculty of Medicine, Thammasat University, Pathum Thani, Thailand; c Division of Nephrology, Department of Internal Medicine, Thammasat University Hospital, Pathum Thani, Thailand; d Division of Nephrology and Hypertension, Department of Medicine, Mayo Clinic, Jacksonville, FL, USA

**Keywords:** Extracorporeal plasma therapy, plasmapheresis, focal segmental glomerulosclerosis, FSGS

## Abstract

**Purpose:**

This study aimed to assess efficacy of extracorporeal plasma therapy (EPT), including plasmapheresis (PE), immunoadsorption (IA), low-density lipoprotein apheresis (LDL-A), and lymphocytapheresis (LCAP) for adult native kidney patients with primary focal segmental glomerulosclerosis (FSGS).

**Methods:**

A literature search was conducted using MEDLINE, EMBASE and Cochrane Databases through August 2022. Studies that reported outcomes of EPT in adult native kidneys with primary FSGS were enrolled.

**Results:**

18 studies with 104 therapy-resistant or refractory primary native FSGS patients were identified. Overall EPT response rate was 56%, with long-term benefit of 46%. Of the 101 non-hemodialysis (HD) patients, 54% achieved remission, with 30% complete remission (CR) and 23% partial remission (PR). Of 31 patients with PE, response rate was 65%; CR and PR rates were 27% and 37% in 30 non-HD patients. Of 61 patients with LDL-A, the response rate was 54%; CR and PR rates were 41% and 3% in 29 non-HD patients. Of 10 patients with IA, response rate was 40%. Of 2 patients with LCAP, 1 achieved CR, and one developed renal failure. All 3 HD patients showed increase in urine output and gradual decrease in urine protein excretion following PE (*n* = 1) or LDL-A (*n* = 2). 2 of 3 HD patients ultimately discontinued dialysis.

**Conclusion:**

EPT with immunosuppressive therapy showed benefit in some patients with refractory primary FSGS, and PE appeared to have a higher response rate.

## Introduction

Focal segmental glomerulosclerosis (FSGS), a histologic lesion of glomerular injury, predominantly results from glomerular podocyte damage *via* a variety of different pathogenic mechanisms [1]. FSGS classically presents with proteinuria and/or nephrotic syndrome. It is characterized by renal biopsy by sclerotic lesions in parts (segmental) of some (focal) glomeruli on light microscopy and variable degrees of foot processes effacement and/or fusion on electronic microscopy [2–5].

Currently, FSGS is divided into primary (idiopathic), secondary, genetic, and unknown forms using a clinicopathologic approach [6,7]. Such classification is critical for determining appropriate therapy. Secondary forms of FSGS include maladaptive FSGS caused by glomerular hyperfiltration (e.g., obesity, hypertension, reflux nephropathy, and nephrectomy), infection-associated FSGS (such as HIV, CMV, EBV, HCV, and Leishmania), and drug-associated FSGS (e.g., pamidronate, lithium and mTOR inhibitors). Besides therapy directed at the specific cause (such as correction of hyperfiltration), elimination of infectious sources, and discontinuation of offensive drugs, the general treatment strategy in secondary FSGS is based on supportive renoprotection such as RAAS blockade with angiotensin-converting enzyme inhibitors (ACEIs) or angiotensin AT(1)-receptor blockers (ARBs) [6,7]. Nonspecific renoprotective therapy is also used in patients with genetic FSGS [6,7]. Typically, genetic FSGS is resistant to corticosteroids; but several patients have been described as being responsive to steroid and calcineurin [7,8]. Conversely, in primary FSGS, corticosteroids and immunosuppressants are the main therapies, and initial responsiveness is often seen. However, treatment resistance and/or frequent relapses are common during the disease course and are associated with poor outcomes [7,9]. As such, novel alternative therapies are needed for therapy-resistant and/or refractory primary native FSGS.

One of the current hypotheses is that primary FSGS is caused by a circulating permeability factor (i.e., plasma derived factor), possibly from extrarenal sources [10]. Therefore, primary FSGS is also known as permeability factor-mediated FSGS. Several molecules have been proposed as pathogenic permeability factors that can cause podocyte injury, including soluble urokinase‑type plasminogen activator receptor (suPAR), cardiotrophin-like cytokine factor 1 (CLCF-1), and anti-CD40 antibody [11,12]. However, validated and convincing evidence of their consistent pathophysiologic role in clinical practice remains unavailable. Extracorporeal plasma therapy (EPT) is thus prescribed based on the above hypothesis to remove potential permeability factors in primary and post-transplant recurrent FSGS [13,14]. The modalities of EPT include plasma exchange or plasmapheresis (PE), immunoadsorption (IA) with protein A or IgG, low-density lipoprotein apheresis (LDL-A), and lymphocytapheresis (LCAP). In recurrent FSGS after kidney transplantation, EPT removal of a permeability factor can dramatically reduce proteinuria and, in some cases, induce a complete remission (CR) [15]. Whether the data from kidney allograft recipients can be translated to those with primary FSGS in native kidneys is unclear. Bosch et al. summarized several individual case reports that showed the promising efficacy of EPT (PE and IA) in treating primary FSGS in native kidneys [16]. However, the benefits of EPT in case series studies are conflicting. In an uncontrolled study consisting of 11 patients with FSGS resistant to steroids and cytotoxic drug (cyclophosphamide), 73% (*n* = 8) of patients obtained either CR or partial remission (PR) after PE therapy [17]. In contrast, another study in 8 patients with steroid-resistant FSGS showed a relatively poor response with only 25% (*n* = 2) of PR after PE, although the stable renal function was notably observed in 4 patients at last follow up (2 with PR and 2 of 6 in nonresponding patients) [18]. Most recently, an updated review about EPT in kidney disease suggests that PE is still the leading EPT, but is increasingly being replaced by more selective extracorporeal treatment such as double-filtration plasmapheresis or IA in some severe conditions, and in the future, IA may replace PE as the preferred EPT for certain diseases due to its higher efficacy and fewer side effects [19].This study aimed to systematically review and summarize the current literatures in order to assess the efficacy of EPT for adult primary native FSGS patients.

## Methods

### Search strategy

A literature search using MEDLINE (1946 to August 2022), EMBASE (1988 to August 2022), Cochrane Central Register of Controlled Trials, and Cochrane Database of Systematic Reviews (inception to August 2022) was independently conducted by two investigators (J.M. and P.K.) to assess the outcomes of EPT for adult biopsy-proven primary FSGS in native kidneys. The search strategy included the terms ‘focal segmental glomerulosclerosis or FSGS’, AND ‘plasma exchange or plasmapheresis or apheresis or LDL-apheresis or lymphocytapheresis or immunoadsorption’ (Online Supplementary Data). A manual search for additional related studies through the references of the included studies was also performed. No language limitation was applied. This systematic review was conducted by the Preferred Reporting Items for Systematic Reviews and Meta-Analysis (PRISMA) statement [20]. The protocol for this systematic review is registered with PROSPERO (International Prospective Register of Systematic Reviews; no. CRD42022357516).

### Selection criteria

Eligible studies were case reports, case series, clinical trials, or observational studies, including cross-sectional, case-control, or cohort studies that reported the EPT outcomes for adult (>18 years) biopsy-proven primary FSGS in native kidneys. Studies had to include the following outcomes: remissions, relapses, degree of proteinuria, or serum creatinine. Exclusion criteria consisted of studies that primarily reported other treatment outcomes, patients comprised of mixed FSGS with other glomerular diseases, or results without subgroup analysis for FSGS alone. Non-English articles were excluded. Remission was determined by the reduction of proteinuria based on each article. Generally, CR was defined as proteinuria of less than 0.3 g per 24 h and PR as a reduction of proteinuria of 0.3 to 3.5 g per 24 h and 50% reduction from baseline. Retrieved articles were independently reviewed for their eligibility by the two investigators (J.M. and P.K.). Discrepancies were resolved by consensus between all authors.

### Data abstraction

A standardized data collection form was used to gather the following data from each included article: study title, author names, publication year, the country where the study was conducted, study type, number of patients, patient gender, age, whether or not underwent kidney biopsy, previous immunosuppressive treatment and its duration prior to EPT, type of EPT (PE, LDL-A, IA, or LCAP), EPT protocol, concomitant treatment with EPT, duration of follow-up, rate of response (CR and PR), outcome description, and serious adverse events.

## Results

### Retrieved articles for systematic review

**Figure 1.**
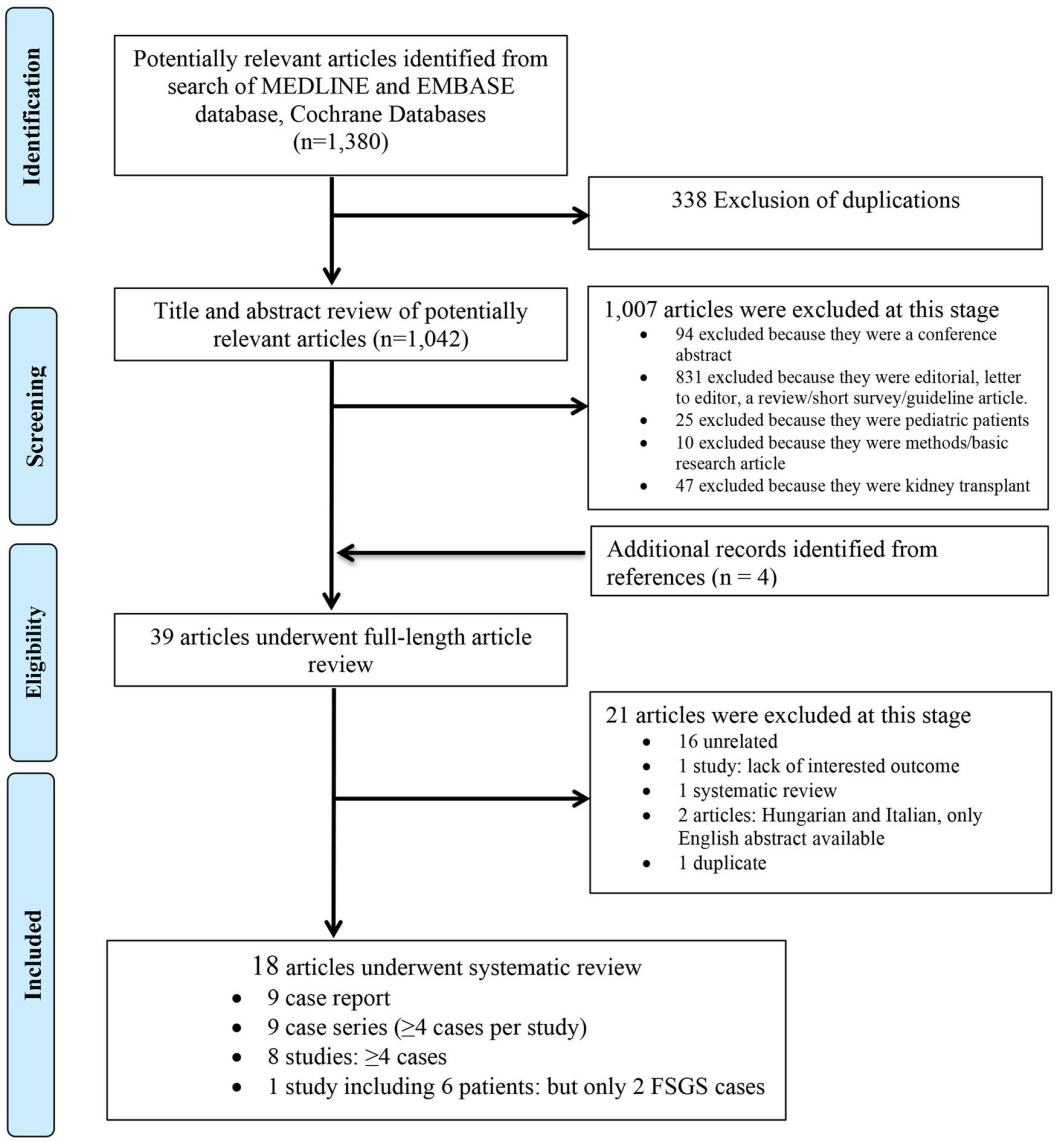
PRISMA flow diagram for study selection.

Our search strategy retrieved a total of 1,380 potentially relevant articles (Figure 1). 338 were duplicated articles. After further exclusion of 1,007 articles because they clearly did not meet inclusion criteria due to article type, methodology, or outcomes of interest, 35 articles were identified. Four additional records were identified from the references of the included studies. Thus, 39 articles underwent full-length review. An additional 21 articles were excluded due to failing to meet inclusion criteria as shown in Figure 1. Ultimately,18 articles were included in the systematic review: 9 case reports [21–29], 8 case series with ≥4 cases per study [17,18,30–35], and 1 case series with only 2 FSGS cases [36]. Of the 9 case series studies, 3 studies included > 10 patients [17,30,34]. Of the 18 studies, only 2 were prospective [30,34].

### Modality and prescription of EPT

A total of 104 patients from 1997 and 2022 were included in this systematic review. PE was reported in 8 studies (5 case reports and 3 case series) (Supplemental Table 1), followed by LDL-A in 6 studies (3 case reports and 3 case series) (Supplemental Table 2), IA in 3 studies (1 case report and 2 case series) (Supplemental Table 3), and LCAP in a case series study (but only 2 FSGS cases) (Supplemental Table 3).

As shown in Table 1, regarding EPT modality, LDL-A was performed in 61 (59%) patients, followed by PE (*n* = 31, 30%), IA (*n* = 10, 10%), and LCAP (*n* = 2, 2%). LDL-A was performed with a median of 9 sessions (IQR 8, 11) over 4.5 weeks (IQR 4, 5.5) in 3 case report studies [21,26,27], 9.6 ± 2.7 sessions in a case series study [34], and an average of 9.6 sessions per patient in another case series study [30]. PE was performed with a median of 6.5 sessions (IQR 4.5, 14.5) over 2 weeks (IQR 2, 19) in 2 case series and 2 case reports [17,18,23,25], 14 sessions (IQR 10, 23) over 6 weeks in a case series [31], and 35 sessions over 2 years and 88 sessions over 18 months in 2 case report studies [28,29], respectively. IA was performed with a median of 10 sessions (IQR 5, 10) over 4 weeks (IQR 1.4, 4) in 3 studies [24,32,33]. LCAP was reported twice in two consecutive weeks in the case series study [36].

**Table 1.** Modality, session and duration of EPT and renal outcomes in primary FSGS.

Modality of EPT	Study, first author, year (reference)	EPT total sessions	EPT duration	Follow up duration	Responders, *n*/total (%)	CR, *n*/total (%)	PR, *n*/total (%)
**PE** **(*n* = 31 patients)**							
5 case reports	Ginsburg, 1997 [28]	35	2 years	12 months	1		1
	Ishii, 2002 [23]	4	2 weeks	6 months	1		1
	Oliverira, 2007 [25]	7	3 weeks	24 months	1	NA	NA
	Cader, 2017 [29]	88	1.5 years	18 months	1	1	
	Schenk, 2017 [22]	NA	NA	12 months	1		1
3 case series	Feld, 1998 [18]	6	2 weeks	29 ± 4 months	2/8 (25) Long term 1/8 (13)		2/8 (25) Long term 1/8 (13)
	Mitwalli, 1998 [17]	17	6 months	27.5 ± 6.3 months	8/11 (73) Long term 6/11 (55)	6/11 (55) (Long term)	2/11 (18)
	Dirim, 2022 [31]	14 (IQR 10, 23)	>6 weeks	17 months (IQR 15, 20)	5/7 (72) Long term 3/7 (43)	1/7 (14) (Long term)	4/7 (58) Long term 2/7 (29)
**LDL-A** **(*n* = 61 patients)**							
3 case reports	Yorioka, 1997 [27]	8	4 weeks	3 months	1		1
	Araki, 2015 [26]	9	4.5 weeks	∼4 months	1	NA	NA
	Yamazaki, 2016 [21]	11	5.5 weeks	3 weeks	1	NA	NA
3 case series	Muso, 2015 [30]	average 9.6	NA	4 weeks	14/26 (54) ^a^	NA	NA
	Muso, 2015 [34]	9.6 ± 2.7	NA	2 years	12/28 (43) (Long term)	12/28 (43) (Long term)	
	Muso, 1999 [35]	NA	NA	2 weeks	4/7 (57)	NA	NA
**IA (*n* = 10 patients)**							
1 case report	Kuhn, 2006 [24]	10	4 weeks	NA	1		1
2 case series	Haas, 1998 [33]	5	10 days	NA	2/5 (40)		2/5 (40)
	Moriconi, 2001 [32]	10	4 weeks	6 months	1/4 (25)		1/4 (25)
**LCAP** **(*n* = 2 patients)**							
1 case series	Yokoyam, 2002 [36]	4	2 weeks	47 and 40 months	1/2 (50)	1/2 (50)	

EPT: extracorporeal plasma therapy; FSGS: focal segmental glomerulosclerosis; CR: complete remission; PR: partial remission; PE: plasmapheresis or plasma exchange using albumin; NA: not available; IQR: interquartile (25% and 75% percentile); LDL-A: LDL apheresis using dextran sulfate cellulose column; IA: immunoadsorption using protein A or IgG; LCAP: lymphocytapheresis using Cellsorba, a leukapheresis filter. **^a^** 26 episodes of LDL-A in 23 patients.

EPT was performed concomitantly with steroids and/or immunosuppressive therapy (such as prednisone, cyclosporine, azathioprine, cyclophosphamide, mycophenolate mofetil, and calcineurin inhibitor) in most studies. In some of the reports, their dosage was tapered and discontinued (Supplemental Table 1–3). There appeared to be no standard protocol for EPT. Most EPT were prescribed at a frequency of twice a week, particularly at the initiation stage, followed by a gradual decrease according to the patient’s response. Two case report studies from Gimsburg et al. and Cader et al. reported a total of 35 and 88 sessions of PE within 1 and 1.5 years, respectively; both patients achieved a long-term partial or complete remission [28,29].

### General characteristics of the patients

In this review, 59 (57%) of the 104 patients did not have biological sex recorded composed of 3 case series [18,30,34] (Table 2). The remaining 45 patients consisted of 42% (*n* = 19) males and 58% (*n* = 26) females. All 104 adult native kidney patients had therapy-resistant or refractory biopsy-proven primary FSGS. The mean age of the 9 case report studies (with total of 11 patients) and a case series that only had 2 FSGS was 39.7 ± 20.3 years (median 34; IQR 24, 45) [21–29,36]. The mean age of 4 case series studies with a total of 30 patients was between 26.4 and 34 years [17,31,33,35]. The age of a case series study with 4 patients ranged from 18 to 60 years [32]. The age of the other 3 case series studies, with a total of 59 patients, was not described [18,30,34].

**Table 2.** General characteristics of patients with primary FSGS.

Patients (*n*)	Male, n/total (%)	Age (y)
Non-hemodialysis (101)	18 M; 24 F	
Hemodialysis (3)	1 M; 2 F	74; 43 and 81
Total (104)	19/45 (42) NA in 3 studies (*n* = 8, 23 and 28)	9 case report studies and a case series study (*n* = 11): 39.7 ± 20.3; 34 (IQR 24, 45) **^a^** 8 case series studies Haas [33]: *n* = 5; 28.4 ± 12.4; 25 (IQR 19.5, 39) Mitwalli [17]: *n* = 11; 32 ± 6.6 Dirim [31]: *n* = 7; 26.4 ± 9.8; 23 (IQR 19, 32) Muso [35]: *n* = 7; 34 ± 13.8; 26 (IQR 22, 46) Moriconi [32]: *n* = 4: range 18–60 NA in 3 case series (*n* = 8, 23 and 28) [18,30,34]

FSGS: focal segmental glomerulosclerosis; M: male; F: female; NA: not available; IQR: interquartile (25% and 75% percentile). **^a^**9 case report studies [21–29]. The case series study only had two cases with FSGS [36].

### EPT Outcomes for adult primary FSGS native kidneys

Overall, 58 (56%) of 104 patients were responders at the end of EPT, and the long-term (follow-up ≥6 months) efficacy rate was 46% (*n* = 30/65) (Table 3). One hundred one non-hemodialysis patients were reported in 15 studies. CR and PR were defined in 5 studies (*n* = 56 patients), though definitions differed [17,18,31,34,36]. In the other 9 studies (*n* = 15 patients), CR and PR were not described and defined; we determined CR and PR by change in proteinuria levels based on its description in the articles [22–24,27–29,32,33]. In these patients, CR was defined as a reduction of baseline proteinuria to urine protein excretion of less than 0.3 g per 24 h and PR as a reduction of baseline proteinuria to urine protein excretion of 0.3 to 3 g per 24 h and 50% reduction in proteinuria. In two studies with 30 patients, only remission or effectiveness were reported [30,35]. Of the 101 non-hemodialysis patients, 54% (*n* = 55) achieved remission after EPT; CR and PR were 30% (*n* = 21/71) and 23% (*n* = 16/71), respectively.

**Table 3.** Overall renal outcomes following EPT therapy in primary FSGS.

Patients (*n*)	Responders, n/total (%)	CR, n/total (%)	PR, n/total (%)
Non-hemodialysis (101)	55/101 (54)	21/71 (30)^a^	16/71 (23)^a^
Hemodialysis (3)	3/3 (100)		
Total (104)	58/104 (56) Long-term 30/65 (46)^b^		

EPT: extracorporeal plasma therapy; FSGS: focal segmental glomerulosclerosis; CR: complete remission; PR: partial remission. ^a^Not available in 2 case series studies (*n* = 7 and 23, respectively). Remission and effectiveness were reported in these 2 studies. ^b^Long-term indicates follow-up duration is ≥6 months.

Outcomes of different EPT modality are shown in Table 4. Of the 31 patients who underwent PE, the response rate was 65% (*n* = 20) in ≥14 months of follow-up duration. CR and PR rates with PE were 27% (*n* = 8) and 37% (*n* = 11) in 30 non-hemodialysis patients, respectively. Of the 61 patients who underwent LDL-A, the response rate was 54% (*n* = 33) but it was primarily reported with a short follow-up period (≤4 months). Only 1 case series study followed 2 years after LDL-A therapy, and the long-term response rate was 43% (*n* = 12/28). CR and PR rates with LDL-A were 41% (*n* = 12) and 3% (*n* = 1) in 29 non-hemodialysis patients, respectively. A significant reduction of LDL and total cholesterol was also reported after LDL-A therapy [27,30,34,35]. Of the 10 patients with IA, the response rate (all presenting with PR) was 40% (*n* = 4) with 6 months of follow-up. Only one study, consisting of 6 patients (2 minimal change disease (MCD), 2 FSGS, 1 membranous nephropathy (MN), and 1 MN and FSGS), reported the efficacy of LCAP in primary kidney disease. LCAP was performed twice in two consecutive weeks and then followed with corticosteroid therapy with or without cyclosporine [36]. Of the 2 patients with FSGS, one achieved complete remission, and the other one did not respond and progressed to renal failure at the end of follow-up (47 and 40 months, respectively). The patients with MN and FSGS obtained PR, another 2 patients with MCD achieved CR, and the patient with MN died from pneumonia after LCAP therapy. T cells (especially activated T cells) decreased significantly after LCAP therapy in the response group [36].

**Table 4.** Summary of renal outcomes following different EPT in primary FSGS.

Patients (*n*)	Responders, n/total (%)	CR, n/total (%)	PR, n/total (%)	Follow up duration
**PE (**31)	20/31 (65) Long-term 15/31 (48)^a^			14.4 ± 6.8 months in 5 case report studies [22,23,25,28,29] 29 ± 4 months in a case series study [18] 27.5 ± 6.3 months in a case series study [17] 17 months (IQR 15, 20) in a case series study [31]
Non-HD (30)	19/30 (63)	8/30 (27)	11/30 (37)	
HD (1)	1/1 (100)			
**LDL-A** (61)	33/61 (54) Long-term 12/28 (43)			3 weeks, 3 months and 4 months in 3 case report studies, respectively [21,26,27] 2 week, 4 weeks and 2 years in 3 case series studies, respectively [30,34,35]
Non-HD (59)	31/59 (53)	12/29 (41)^b^	1/29 (3)^b^	
HD (2)	2/2 (100)			
**IA** (10)^c^	4/10 (40) Long-term ¼ (25)	0/10 (0)	4/10 (40)	25 weeks and 6 months in 2 patients, respectively [32] NA in 7 patients
**LCAP** (2)^c^	1/2 (50) Long-term 1/2 (50)	1/2 (50)		47 and 40 months, respectively [36]

EPT: extracorporeal plasma therapy; FSGS: focal segmental glomerulosclerosis; CR: complete remission; PR: partial remission; IQR: interquartile (25% and 75% percentile); PE: plasmapheresis or plasma exchange using albumin; HD: hemodialysis; LDL-A: LDL apheresis using dextran sulfate cellulose column; IA: immunoadsorption using protein A or IgG; NA: not available; LCAP: lymphocytapheresis using Cellsorba, a leukapheresis filter.

^a^Long-term indicates follow-up duration is ≥6 months. ^b^NA in 2 case series studies (*n* = 7 and 23, respectively). Remission and effectiveness were reported in the 2 studies [30,35]. ^c^The two patients are non-hemodialysis.

Regarding EPT therapy on dialysis patients, three studies reported 3 hemodialysis patients caused by FSGS who received PE (*n* = 1) and LDL-A (*n* = 2) [21,25,26]. All 3 patients showed an immediate increase in urine output and a gradual decrease in urine protein excretion after EPT. Of note, 2 patients ultimately discontinued hemodialysis after 3 and 5 months, respectively. Of the 2 patients, 1 remained off dialysis with stable renal function even two years later, and the other patient no longer required dialysis during at least 40 days follow-up period [25,26]. The third hemodialysis patient was an 81-year-old female with idiopathic collapsing FSGS who underwent LDL-A. Her urine volume gradually increased from anuria to 150 mL/day, but she was unable to be liberated from hemodialysis and ultimately died three months later due to general condition deterioration [21].

## Discussion

Primary FSGS, one of the major causes of nephrotic syndrome (NS), occurs in approximately 20% of adults, and the underlying etiology is often unknown [37–40]. A circulating factor is thought to play a crucial role in the pathophysiology of increased glomerular filter permeability by the rapid and extensive podocyte foot process effacement and subsequent proteinuria development in primary FSGS. Besides RAS blockade and other renal supportive therapies, corticosteroids and immunosuppressants are the main treatments for primary FSGS. However, due to frequent relapses and/or drug resistance, refractory FSGS is not uncommon, and end-stage kidney disease develops in approximately 30% of affected patients after 10 years [41] and in nearly all patients with massive proteinuria within 5 years [40]. Some studies show that EPT may be a promising therapeutic strategy for primary FSGS. However, the currently available literature is conflicting, and convincing evidence remains unavailable due to the lack of prospective, randomized clinical trials. This study systematically summarized and assessed the available current data regarding the efficacy of EPT for adult native kidney patients with primary FSGS. Nine case reports included in this study, consisting of 5 PE, 3 LDL-A, and 1 IA, reported good treatment response at the end of EPT [21–29]. But the results between different case series studies were remarkably variable. Consequently, the overall response rate after EPT was 56%. Furthermore, the long-term (follow-up ≥ 6 months) efficacy rate was only 46%.

One EPT is PE, which typically utilizes albumin as replacement fluid, although fresh frozen plasma may be used in select cases. In 1998, Mitwalli et al. reported that in 11 patients with therapy-resistant (prednisone alone or with cyclophosphamide) primary FSGS, 73% (*n* = 8) achieved remission (6 persistent CR and 2 temporary PR) after a total of 17 PE sessions over a 6-month period; the average follow-up duration was 27.5 ± 6.3 months [21]. Meanwhile, Feld et al. reported poor efficacy of PE during a similar follow-up period (29 ± 4 months) in 8 patients with steroid-resistant idiopathic FSGS; only 25% (*n* = 2) obtained PR, and none achieved CR after 6 sessions of PE within 2 weeks [18]. The big difference between these two studies is the number of PE sessions (17 vs 6 sessions). In a case report of a 22-year-old female with early-stage primary FSGS who failed to respond to intensive immunosuppressive therapy, Gineburg & Dau showed an immediate reduction of serum creatinine from 2.9 to 1.6 mg/dl and an increase in serum albumin from 3 to 3.7 g/dl after 1 week of PE, and a reduction of proteinuria by >50% from 8.8 to 2 g/day after 1 month of PE (total therapy consisted of once weekly for 2 months). One year later, after a total of 35 sessions of PE, her urine protein remained at 1.6 g/day, her creatinine clearance was 90 mL/min, and serum creatinine was 1.2 g/dl [28]. In another case report of a 24-year-old female with therapy-resistant (corticosteroids, mycophenolic acid, and rituximab) primary FSGS, Cader et al. showed a remarkable reduction of UPCR from an average of 0.5–0.8 g/mmol to 0.05–0.08 g/mmol, an increase in serum albumin level from <2 g/dl to 3.8 g/dl, and a persistently stable serum creatinine of 1.13 mg/dl after a total of 88 sessions of PE over approximately 17 months [29]. These findings suggest that prolonged PE may be required to achieve clinical response in FSGS patients. Notably, PE non-selectively removes plasma components from blood, such as antibodies, immune complexes, inflammatory mediators, albumin, and other useful components in the plasma, and returns red blood cells and platelets in plasma or a plasma-replacing fluid [19]. Therefore, this procedure will lower or suppress the body’s immunity and also increases the risk of transfusion-related infections or allergic reactions [19]; for this reason, 5% albumin is preferred to fresh frozen plasma (FFP) because it causes fewer reactions and transmits no infections.

Immunosuppressants were concomitantly used during PE therapy in all studies. Alkylating therapy (cyclophosphamide) was reported during PE in the study of Mitwalli [17]. Dirim et al. reported similar efficacy of PE in 7 patients with refractory primary FSGS as described by Mitwalli, showing that 72% (*n* = 5) achieved remission (1 CR and 4 PR) after 14 sessions (IQR 10, 23) over at least 6 weeks with a median of 17 months (IQR 15, 20) follow-up period [31]. The long-term rate (43%) in the case series of Dirim et al. was obviously better than that (25%) in Feld et al. study [18], but lower than that (55%) in the study of Mitwalli. Cyclosporine, not cyclophosphamide, was used during PE in the case series of Dirim et al. Similarly, the study of Feld et al. did not use alkylating therapy and other immunosuppressants. Therefore, concomitant use of immunosuppressants in particular alkylating therapy may help improve the efficacy of PE. However, its side effects, such as infertility, myelosuppression, and malignancy, limit its application [42].

LDL-A was another frequently used modality of EPT though its therapeutic mechanism of action is not well known yet. This study compiled a large number of adult primary FSGS who received LDL-A. Of the 61 patients with LDL-A therapy in 3 case reports and 3 case series studies, the response rate was 54% (*n* = 33) after about 10 sessions within approximately one month. In a case series study consisting of 8 patients (7 FSGS and 1 MCD) who failed full dose corticosteroids after ≥1 month, Muso et al. showed 57% (*n* = 4/7 FSGS) of remission (daily proteinuria <3.5 g/day and serum albumin >3 g/dl) two weeks after LDL-A therapy [35]. In 2015, Muso et al. additionally reported two case series studies for drug-resistant NS treated with LDL-A therapy that consisted of prospective trials (POLARIS study) with short- and long-term outcomes [30,34]. The short-term POLARIS study consisted of 44 patients (23 primary FSGS, 4 MN, 3 Henoch-SchÖnlein purpura nephritis, 2 MCD, 2 renal amyloidosis, 5 assorted etiologies (membranoproliferative glomerulonephritis, crescentic glomerulonephritis, IgA nephropathy, lupus nephropathy, hepatitis B virus-associated nephropathy), and 5 uncertain. The clinical efficacy of LDL-A was evaluated using the 24-h proteinuria level at the completion of treatment. The results showed an efficacy rate of 54% in 23 primary FSGS patients and a similar efficacy rate of 50% (8/16 patients) for other glomerular diseases [30]. In the long-term POLARIS study, consisting of 44 patients (28 primary FSGS, 4 MN, 2 MCD, 2 renal amyloidosis, 2 undescribed cases, 1 lupus nephritis, 1 membranoproliferative glomerulonephritis, 1 Henoch-Schönlein purpura nephritis, 1 crescentic glomerulonephritis, 1 diabetic nephropathy, and 1 hepatitis B virus-associated nephropathy), 21 (48%) patients showed remission of NS based on a proteinuria level <1 g/day. Of the 28 drug-resistant primary FSGS, 12 (43%) showed remission after 2 years of LDL-A treatment [34]. However, an uncontrolled prospective case series Sweden study published in 2000 consisting of 7 patients with MCD, MN, and IgA nephropathy conversely showed that LDL-A therapy was ineffective in reducing nephrotic range proteinuria even though a small increase in serum albumin and a significant decrease in serum cholesterol, apo B and plasma Lp9a levels were noted [43]. In another case series with 41 refractory FSGS (including 7 cases in transplant kidneys) showed remission (CR or type I incomplete remission defined by proteinuria negative or <1 g/day and serum albumin >3 g/dl) in 62% (18/29) patients at 2 years and 86% at 5 years after 3–12 (8.3 ± 2.9) sessions of LDL-A treatment combined with steroids (*n* = 34) and/or cyclosporine (*n* = 8), and other immunosuppressive drugs (*n* = 8) [44]. These studies all observed a significant reduction in LDL and lipoproteins (e.g., total cholesterol) after LDL-A therapy. It should be noted that all LDL-A studies in adult primary FSGS were conducted in the Japanese population.

IA is another modality of EPT that is more efficient at removal of immunoglobulins than PE [45]. Protein A and sheep anti-human polyclonal IgG antibodies are most frequently used in IA to eliminate IgG. There are only two case series studies and one case report consisting of 10 patients that reported IA for renal-directed therapy. Of the 10 patients, the rate of response (all PR) was 40% (*n* = 4) after IA treatment. In a case series study, consisting of 5 patients with refractory FSGS who were resistant to corticosteroids, cyclophosphamide, and cyclosporine, and underwent IA (*n* = 4) or IgG-IA (*n* = 1) for at least 5 sessions for 10 days, Haas et al. showed that only 2 patients achieved PR [33]. Another case series study, consisting of 4 patients with therapy-resistant FSGS (2 early FSGS, 1 late-stage FSGS, and 1 partially advanced FSGS), reported minimal efficacy after at least 10 sessions of IA. In this particular study, the only patient that had a clinical remission (normalization of permeability factor and serum creatinine levels and an apparent decrease in proteinuria from 14 to 4 g/day at week 8 and to 2 g/day at week 25) was the one with partially advanced FSGS; the other 3 patients did not respond to IA treatment [32]. In 2006, a case report of a 34-year-old male with therapy-resistant (corticosteroids, cyclophosphamide, cyclosporine, tacrolimus, and mycophenolate mofetil) primary FSGS described PR (a reduction of proteinuria from 9.2 to 4.5 g/day) with a constant increase in serum albumin and reduction in serum cholesterol during IA [24]. Two months after IA, though proteinuria relapsed to 19 g/day, PR (a reduction of proteinuria by >50% from 19 to 3 g/day) was re-achieved after continued IA therapy (2 sessions a week for 4 weeks followed by once a week without immunosuppressants) [24]. Conversely, a different case report did not show any response of IA despite the concomitant use of PE in a 34-year-old female with therapy-resistant FSGS whose proteinuria relapsed after a successful treatment of PE [22]. Hence, the efficacy of IA in refractory primary FSGS and who it should be targeted to remain unclear due to limited data.

The application of LCAP has been described in many patients with lymphocyte abnormalities, such as rheumatoid arthritis [46,47]. Glomerular permeability factors derived from T lymphocytes may play a critical role in podocyte injury and proteinuria development in patients with NS, especially MCD [48]. In native primary FSGS or recurrent FSGS after renal transplantation, a circulating glomerular permeability factor has been detected [10,49]. Therefore, LCAP may improve renal outcomes in patients with MCD and FSGS by reducing circulating lymphocytes, particularly T cells. So far, only one study consisting of 2 patients with primary FSGS reported LCAP efficacy [36]. Of the 2 patients, one achieved CR, and the other one did not have a response, with subsequent end-stage renal failure at the end of the follow-up. The efficacy of LCAP in primary FSGS needs to be further studied due to the different effects and very limited patient numbers.

It should be noted that following PE (*n* = 1) and LDL-A (*n* = 2) therapy, all 3 hemodialysis patients due to primary FSGS reviewed here showed an immediate increase in urine output and a gradual decrease in urine protein excretion, and 2 patients ultimately discontinued hemodialysis [21,25,26]. A recent study, including 21 patients with refractory and steroid-dependent NS (12 primary FSGS and 9 MCD), reported PE in 13 patients, IA in 6 patients, PE in conjunction with IA in 1 patient, and LDL-A in 1 patient. This study showed that 3 of 7 patients (43%) who required hemodialysis at the beginning of the procedure achieved remission during follow-up [50].

In addition, there was a comparative study (K-FLAT) that compared the efficacy of LDL-A with steroids monotherapy in steroid-resistant NS patients (LDL-A group: 14 FSGS and 3 MCD vs. steroid group: 9 FSGS and 1 MCD) [51]. In this study, LDL-A was performed with a fixed protocol (twice a week for 3 weeks followed by weekly for 6 weeks). The reduction of serum cholesterol and phospholipid levels were detected only in the LDL-A group. The LDL-A group also showed a significant decrease in proteinuria level and an increase in serum albumin. The percentage of patients achieving both short- and long-term remission was higher in the LDL-A group than in the steroid monotherapy (71% vs. 30% and 88% vs. 44%, respectively). This strongly suggests that improvement of hypercholesterolemia by LDL-A in steroid-resistant NS may provide more relief from NS than steroid therapy alone. Nevertheless, a comparative study remains lacking in patients only with primary FSGS.

It has been proposed that LDL-A can ameliorate glomerular lipotoxicity by decreasing LDL and improving macrophage functions [31], and IA is more potent in removing immunoglobulins than PE. It is also possible that LDL-A can remove some circulating permeability factors with the lipophilic nature. However, other plasma proteins, such as fibrinogen, are cleared more efficaciously in PE [52]. Our current analysis showed higher efficacy of PE than LDL-A and IA (65% vs. 54% and 40%), suggesting that PE appeared to be a promising option in adult patients with refractory primary FSGS. However, it has been noted above that data is limited, and there are some confounding variables (e.g., patients’ different age groups, sex, nationalities, immunosuppressants use, and severity of NS) and statistical analysis is not unavailable to show if there is statistical significance. As such, a prospective, randomized, comparative study of these modalities for the treatment of refractory primary FSGS is needed.

Finally, it should be pointed out that the current systematic review has some limitations. Firstly, the studies reviewed here span a long time period from 1997 to present. As such, more recent studies that use modern care including new medications may influence the efficacy of EPT. Secondly, genetic studies may not have been performed in these studies, or if performed, may not have had the same capabilities that we have today, as genetic FSGS may respond less well to therapy. Thirdly, there were no controlled or comparative randomized clinical trials; only 2 studies were prospective. The study population should also be considered; almost all LDL-A studies were reported in Japanese people.

## Conclusion

EPT utilized concomitantly with immunosuppressive therapy showed benefit in some patients with refractory primary FSGS. PE appeared to have a higher overall response rate than LDL-A and IA; however, large prospective, multi-center, randomized clinical studies are needed to determine the best protocol of EPT (i.e., modality, session, and duration) and concomitant immunosuppressive drugs during EPT therapy and the patients most likely to respond to it.

## Supplementary Material

Supplemental Material

Supplemental Material

Supplemental Material
