# Supplementary material for: Efficacy of extracorporeal plasma therapy for adult native kidney patients with Primary FSGS: a Systematic review
Source: Ren Fail. 2023 Feb 10;45(1):2176694. doi: 10.1080/0886022X.2023.2176694 (PMC9930861; doi:10.1080/0886022X.2023.2176694)
Supplement: Supplemental Material [file IRNF_A_2176694_SM0918.pdf]

Supplemental Table 1 Characteristics of included studies: PE

Study, first author	Year	Study type	Patients, n	Sex	Age (y)	Primary FSGS	Biopsy	Previous treatment	Duration of treatment prior to EPT	EPT protocol	Concomitant treatment	Duration of follow-up	Responder, n/total (%)[a]	CR, n/total (%)[a]	PR, n/total (%)[a]	Outcome description [b]
Ginsburg DS & Dau P	1997	Case report	1 (Caucasian)	F	22	yes	yes	Intensive IS therapy (prednisone, AZA, and CP)	1 year	PE, once a week; the 1st period: 2 months (**8 sessions**); the 2nd period: 5 months (**10 sessions**); the 3rd period: 1 year (gradually increased interval to 3 weeks; **17 sessions**)	Moderate dose of prednisone (30 mg/day) and AZA (200 mg/day); CsA 2mg/kg during the 3rd period	12 months	1 (long-term)		1	• PR during the 1st period (a reduction of Cr from 2.9 to 1.6 mg/dl and increase of albumin from 3 to 3.7 g/dL after 1 week, a reduction of proteinuria by >50% from 8.8 to 2 g and increase of Cr clearance from 24 to 95 ml/min after 1 month)<br>• PR during the 3rd period (immediate decrease in UPCR from 10 to 2, urine protein 1.6 g/day and CC 90 ml/min, SCr 1.2 g/dl after 1 year)<br>• A marked decrease in the B-cell population (CD19+, CD19+CD23+, CD19+CD5), and an increase both in T-lymphocytes of the mature CD4+ helper/suppressor phenotype (CD4+CD45RO+) and in the immature (CD8+CD45RA+) or cytotoxic (CD8+CD28+) CD8+ cells after 8-15 months treatment
Feld et al.	1998	Case series	8 (3 Hispanic, 3 African American, 2 Caucasian)	NA	NA	yes	yes	At least 40 mg prednisone/day for at least 2 months	2 months	PE, **6 sessions** over ~2 weeks	Prednisone	29±4 months	2/8 (25%)<br><br>1/8 (12.5%) (**Long-term**)		2/8 (25%)	• PR in 2/8 (a persistent remission at least 8 months and a brief remission for 2 months, respectively) [c]<br>• A stable renal function at last follow-up in 4/8, including 2 patients with PR and 2 of the 6 nonresponding patients; 4 of the 6 nonresponding patients had significant progression of kidney function and required dialysis treatment

Information Classification: General

Mitwalli et al.	1998	Case series	11	5M 6F	32 ± 6.6	yes	yes	All patients: oral PSL 1–2 mg/kg for a period of 3–4 months then tapered over 2 months to reach 10 mg daily; 7 patients: plus i.v. CP 5–10 mg/kg monthly for 3 doses then every 2 months for a total of 10 doses	Steroids 10-15 months (mean 11.4±1.7)	PE: a total of **17 sessions** over a 6-month period, five consecutive sessions daily in the first week, then twice a week for 2 weeks, then once a week for 2 weeks, then once every 2 weeks for 4 weeks, and then monthly for 4 months	PSL 60–80 mg daily orally for 2 months tapered gradually to 10 mg p.o. daily over the ensuing 4-month period, and CP 5–10 mg/kg i.v. monthly for 6 months	27.5 ± 6.3 months (range 12-37)	8/11 (73%)<br><br>Long-term 6/11 (55%)	6/11 (55%)	2/11 (18%)	• Remission in 8/11 (6 CR and 2 PR) [d]: 6 patients showing a long-term CR and another 2 showing a temporary PR after 1 month and stable renal function
Ishii E et al.	2001	Case report	1 (Japanese)	M	45	yes	yes	Oral PSL 30mg/day or plus mizoribine (150 mg/day)	3 years	PE, **4 sessions** over 2 weeks	Oral PSL 30mg/day or plus mizoribine (150 mg/day)	6 months	1 (long-term)		1	• A marked reduction of urinary protein within 1 week.<br>• PR (a reduction of proteinuria by >50% from 3.2 to 0.6 g/day at the end of PE therapy)<br>• Proteinuria remained suppressed for > 6 months despite a reduction of PSL dose to 15 mg
Oliverira et al.	2007	Case report	1	M	74	yes	yes	Progressive to ESRD and HD was started. No response to prednisone 80 mg/day (1mg/kg/day) over 3 months	3 months	PE; a total of **7 sessions** over a 3-week period	Prednisone 40mg/day plus oral CP 75 mg/day (1mg/kg/day)	2 years	1 (long-term)	NA	NA	• Urine output increase<br>• Discontinued HD 5 months later<br>• Remained off dialysis with stable renal function (creatinine clearance: 40 mL/min) two years later
Cader et al.	2017	Case report	1 (Chinese)	F	24	yes	yes	CS, mycophen	6 years	PE, 3 sessions per week	Prednisone,	1.5 years	1 (long-term)	1		• CR (a remarkable reduction of UPCR from an average of 1.5-0.8 to 0.05-0.08 g/mmol

Information Classification: General

								olic acid and RTX		within 3 months (**36 sessions**), tapered to twice a week for 2 months (**16 sessions**) later to once a week for 6 months (**24 sessions**), then remained once per two weeks for ~6 months (**12 sessions**)	MMF, Tac					with serum albumin level from < 20 g/L previously to current level of 38 g/L, with a stable Cr of 100 µmol/L)
Schenk et al.	2017	Case report	1	F	34	yes	yes	CS, CsA	CS > 3 months	PE, NA in sessions	RTX	1 year	1 (long-term)		1	• PR (a reduction in proteinuria from 28 to 13 g/g creatinine with a stable creatinine level)<br>• Over the following 2 years, not adherence to IS (MMF) and supportive (ACEI) therapy led to increase of Cr and proteinuria. Then **PE and IA** were initiated, but no response. **CytoSorb** was started, showing reduction of suPAR. But finally, HD was required.
Dirim et al (#152)	2022	Case series	7	2M 5F	Median age 23 (IQR 19, 32)	yes	yes	All patients: RAS blockade, CS and CNI Most of patients: at least one of the other IS (RTX, mycophenolate, levamisole, or CP)	From diagnosis to the 1st PE: median 24 months (IQR 24, 96)	PE; median **14 session (IQR 10, 23)**; 3 sessions per week for 3 weeks. Then, 2 sessions per week for 3 weeks. If need additional PE: 1	CsA, RAS blockade, methyl prednisolone followed by PSL; RTX after the last PE in selected RTX	17 months (IQR 15, 20)	5/7 (72%); Long-term 3/7 (43%)	1/7 (14%)	4/7 (58%)	• Remission in 5/7 (1 CR and 4 PR) [e]<br>• Sustained remission during follow-up in 3/7 (1 CR and 2 PR)

Information Classification: General

										session per week until month three, 2 sessions per month until month five, and 1 session per month until month nine	naïve patients					

Abbreviations: PE: plasmapheresis or plasma exchange using albumin; FSGS: focal segmental glomerulosclerosis; CR: complete remission; PR: partial remission; M: male; F: female; NA: not available; AZA: azathioprine; CP: cyclophosphamide; SCr: serum creatinine; CsA: cyclosporine; PSL: prednisolone; IS: immunosuppressive or immunosuppressants; CS: corticosteroids; Tac: tacrolimus; RTX: rituximab; MMF: mycophenolate mofetil; ACEI; angiotensin-converting enzyme inhibitors; CNI: calcineurin inhibitor; Pulse: methylprednisolone pulse therapy; IA: immunoadsorption using protein A; HD: hemodialysis; ESRD: end stage renal disease.

[a] The percentage of responder, CR and PR is shown for case series studies.

[b] If not mentioned in the studies, CR and PR is determined by the alteration of proteinuria. CR is defined as reduction of proteinuria to protein excretion less than 0.3 g per 24 hours and PR as reduction of proteinuria to protein excretion of 0.3 to 3 g per 24 hours and 50% reduction in proteinuria from baseline.

[c] CR was defined as a decline in proteinuria to less than 0.5 g/24 h/1.73 m2 and PR by a decline in proteinuria to less than 50% of the baseline value measured within 1 week after PE.

[d] CR: a reduction of urine protein excretion to 0.5 g/24 h or less, with serum creatinine <200 mmol/L. PR: non-nephrotic proteinuria persisted in the absence of hypo-albuminemia (defined as less than 2.5 g/L) and of chronic renal insufficiency.

[e] CR: eGFR >60 ml/min/1.73 m2 and UPCR<0.5 g/g. PR: the >50% reduction of UPCR 1 week after the last PE (Also, serum albumin level should be >2.5 g/L).

References

1. Ginsburg DS, Dau P. Plasmapheresis in the treatment of steroid-resistant focal segmental glomerulosclerosis. Clin Nephrol. 1997;48(5):282-7.
2. Feld SM, Figueroa P, Savin V, Nast CC, Sharma R, Sharma M, et al. Plasmapheresis in the treatment of steroid-resistant focal segmental glomerulosclerosis in native kidneys. Am J Kidney Dis. 1998;32(2):230-7.
3. Mitwalli AH. Adding plasmapheresis to corticosteroids and alkylating agents: does it benefit patients with focal segmental glomerulosclerosis? Nephrol Dial Transplant. 1998;13(6):1524-8.
4. Ishii E, Ando Y, Tamba K, Masunaga Y, Kusano E, Asano Y. Rapid and persistent reduction of proteinuria following plasma exchange in a case of steroid-resistant focal segmental glomerulosclerosis. Ther Apher. 2002;6(2):174-7.
5. Oliveira L, Wang D, McCormick BB. A case report of plasmapheresis and cyclophosphamide for steroid-resistant focal segmental glomerulosclerosis: recovery of renal function after five months on dialysis. Ther Apher Dial. 2007;11(3):227-31.
6. Cader RA, Kang TM. Plasmapheresis in refractory FSGS. J Clin Nephrol Res. 2017;4(4):1072.

Information Classification: General

7. Schenk H, Muller-Deile J, Schmitt R, Brasen JH, Haller H, Schiffer M. Removal of focal segmental glomerulosclerosis (FSGS) factor suPAR using CytoSorb. J Clin Apher. 2017;32(6):444-52.
8. Dirim AB, Demir E, Guller N, Safak S, Artan AS, Oto OA, et al. Efficacy of intravenous combined immunosuppression with plasmapheresis in adult patients with refractory primary focal segmental glomerulosclerosis. J Clin Apher. 2022;37(4):376-87.

Information Classification: General
