# Supplementary material for: Efficacy of extracorporeal plasma therapy for adult native kidney patients with Primary FSGS: a Systematic review
Source: Ren Fail. 2023 Feb 10;45(1):2176694. doi: 10.1080/0886022X.2023.2176694 (PMC9930861; doi:10.1080/0886022X.2023.2176694)
Supplement: Supplemental Material [file IRNF_A_2176694_SM0917.pdf]

Supplemental Table 2 **Characteristics of included studies: LDL-A**

Study, first author	Year	Study type	Patients, n	Sex	Age (y)	Primary FSGS	biopsy	Previous treatment	Duration of treatment prior to LDL-A	LDL-A protocol	Concomitant treatment	Duration of follow-up	Responder, n/total (%) [a]	CR, n/total (%) [a]	PR, n/total (%) [a]	Outcomes [b]
Yorioka et al.	1997	Case reports	1 (Japanese)	F	22	yes	yes	Steroids and IS	7 years	LDL-A, twice a week for 4 weeks (**8 sessions**)	Oral PSL 40 mg/day plus CsA 150 mg/day for 4 weeks, then gradually reduced	3 months	1		1	• PR (daily proteinuria declined from 6 g to 2 g after 1 month and to 0.5 g after 3 months; serum albumin increased from 1.4 g /dL to 2.9 and 3.4 g/dL within 1 and 3 months, respectively)<br>• Reduction in SCr and increase in creatinine clearance from 43.1 to 66.9 ml/min<br>• Reduction in total cholesterol and serum lipoprotein(a) after 1 and 3 months, respectively.
Muso et al.	1999	Case series (K-FLAT study)	8 (**7 FSGS**, 1 MCNS) (Japanese)	4M 3F	26 (IQR 22, 46)	yes	yes	Full-dose CS	≥ 1 month	LDL-A, not fixed-protocol	Full-dose CS	2 weeks	4/7 (57%)	NA	NA	• Remission in 5/8 (62.5%), stationary NS in 3/8 (37.5%)<br>• Remission in 4/7 FSGS (57.1%), stationary NS in 3/7 FSGS (43%) [c]<br>• Significant decreases in serum cholesterol and phospholipid levels
Araki et al.	2015	Case report	1 (Japanese)	F	43	yes	yes	1st biopsy: MCD and ATN, starting PSL 40 mg/day and HD; but no improvement in renal function after 65 days; 2nd biopsy: a tip	2 months	LDL-A; twice a week, a total of **9 sessions**	A gradual reduction in PSL after urine volume recovery and followed by CsA 50 mg/day	~4 months	1	NA	NA	• Urine volume gradually increased and urinary protein excretion gradually decreased from 3.5 to 3 g/dL after 6 sessions<br>• Discontinued HD after 9 sessions, 97 days after it was initiated<br>• 140 days after admission, urine protein 1.1 g/day, SCr 0.96 mg/dL, eGFR 50.9 mL/min/1.73m2, and serum albumin 3.2 mg/dL

Information Classification: General

								variant of FSGS								
Muso et al.	2015a	Case series (**POLARIS,** prospective trail, short-term outcome)	44 (**23 primary FSGS**) (Japanese)	27M 17F (NA for FSGS)	55.4 ±17.3 (rang 18-84) (NA for FSGS)	Yes (3 patients complicated with other renal diseases (2 MN and 1 DN)	yes	Full-dose steroids or saturated CsA	At least 4 weeks	LDL-A**;** total **47 sessions,** average 9.6 sessions per patient	Steroids and/or CsA	4 weeks	14/26 (53.8%)	NA	NA	• Very effective (21.3%, n=10) and effective (31.9%, n=15) in 25/47 sessions (53.1%) 4 weeks after a completion of LDL-A treatment<br>• Clinically effective in 14/26 sessions (53.8%) in cases with primary FSGS [d]<br>• Remarkable reduction of urinary protein from 6.5±3 to 3.3±3 g/day in primary FSGS<br>• A significant reduction of LDL and total cholesterol
Muso et al.	2015b	Case series (**POLARIS,** prospective trail, long-term outcome)	44 (**28 FSGS**) (Japanese)	NA	NA	yes	yes	Full-dose steroids or saturated CsA	At least 4 weeks	LDL-A, 9.6±2.7 sessions	Steroid pulse therapy and/or CsA	2 years	12/28 (43%) **(long-term)**	12/28 (43%)		• Overall, 21/44 (47.7%) showed remission (CR and IR I) [e]<br>• Favorite outcome (CR and IR I) in 12/28 FSGS (42.9%) after 2 years of treatment [e]<br>• The UP level immediately after LDL-A and the rates of improvement of serum albumin, eGFR, UP, and total and LDL cholesterol significantly affected the outcome
Yamazaki et al.	2016	Case report	1 (Japanese)	F	81	yes	yes	HD; PSL 0.8 mg/kg/day and CsA 2 mg/kg/day; but urine output failed to increase	40 days	LDL-A; twice a week and a total of **11 sessions**	PSL 0.8 mg/kg/day and CsA 2 mg/kg/day; but urine output failed to increase	3 weeks	1	NA	NA	• After 6 sessions, urine output gradually and slightly increased from anuria to 150 ml/day but unable to withdraw from HD<br>• Died 3 months later due to general condition deterioration

Abbreviations: *LDL-A: LDL apheresis using dextran sulfate cellulose column; M: male; F: female; CR: complete remission; PR: partial remission; SCr: serum creatinine; IS: immunosuppressive or immunosuppressants; CS: corticosteroids; CsA: cyclosporine; PSL: prednisolone; NA: not available; HD: hemodialysis; MCD: minimal change disease; ATN: acute tubular necrosis; UP: urine protein.*

Information Classification: General

[a] The percentage of responders, CR and PR is shown for case series studies.

[b] If not mentioned in the studies, CR and PR is determined by the alteration of proteinuria. CR is defined as reduction of proteinuria to protein excretion less than 0.3 g per 24 hours and PR as reduction of proteinuria to protein excretion of 0.3 to 3 g per 24 hours and 50% reduction in proteinuria from baseline.

[c] Definition: Remission, daily proteinuria <3.5 g/day and serum albumin >3.0 g/dl; incomplete remission, daily proteinuria <3.5 g/day but serum albumin <3.0 g/dl; stationary NS, daily proteinuria <3.5 g/day and serum albumin <3.0 g/dl.

[d] Definition: very effective, the UP level before LDL-A was ≥3.5 g/day and was reduced to <1.0 g/day after LDL-A; effective, the UP level before LDL-A was ≥3.5 g/day and was reduced to <3.5 g/day (but ≥1.0 g/day) after LDL-A, or the UP level before LDL-A was <3.5 g/day (but ≥1.0 g/day) and was reduced to <1.0 g/day after LDL-A; noneffective, cases other than those in which LDL-A was judged to be very effective or effective.

[e] Definition: complete remission (CR)= UP undetectable; incomplete remission (IR) I = UP <1.0 g/day; IR II = UP ⩾ 1.0 to <3.5 g/day, and no effect = UP ⩾ 3.5 g/day.

References

1. Yorioka N, Taniguchi Y, Nishida Y, Okushin S, Amimoto D, Yamakido M. Low-density lipoprotein apheresis for focal glomerular sclerosis. Ther Apher. 1997;1(4):370-1.
2. Muso E, Mune M, Fujii Y, Imai E, Ueda N, Hatta K, et al. Low density lipoprotein apheresis therapy for steroid-resistant nephrotic syndrome. Kansai-FGS-Apheresis Treatment (K-FLAT) Study Group. Kidney Int Suppl. 1999;71:S122-5.
3. Araki H, Ono S, Nishizawa Y, Deji N, Nakazawa J, Morita Y, et al. Focal Segmental Glomerular Sclerosis Ameliorated by Long-term Hemodialysis Therapy with Low-density Lipoprotein Apheresis. Intern Med. 2015;54(17):2213-7.
4. Muso E, Mune M, Hirano T, Hattori M, Kimura K, Watanabe T, et al. Immediate therapeutic efficacy of low-density lipoprotein apheresis for drug-resistant nephrotic syndrome: evidence from the short-term results from the POLARIS Study. Clin Exp Nephrol. 2015a;19(3):379-86.
5. Muso E, Mune M, Hirano T, Hattori M, Kimura K, Watanabe T, et al. A Prospective Observational Survey on the Long-Term Effect of LDL Apheresis on Drug-Resistant Nephrotic Syndrome. Nephron Extra. 2015b;5(2):58-66.
6. Yamazaki J, Kanehisa E, Yamaguchi W, Kumagai J, Nagahama K, Fujisawa H. Idiopathic collapsing focal segmental glomerulosclerosis in an 81-year-old Japanese woman: a case report and review of the literature. CEN Case Rep. 2016;5(2):197-202.

Information Classification: General
