# Supplementary material for: Efficacy of extracorporeal plasma therapy for adult native kidney patients with Primary FSGS: a Systematic review
Source: Ren Fail. 2023 Feb 10;45(1):2176694. doi: 10.1080/0886022X.2023.2176694 (PMC9930861; doi:10.1080/0886022X.2023.2176694)
Supplement: Supplemental Material [file IRNF_A_2176694_SM0916.pdf]

Supplemental Table 3 **Characteristics of included studies: IA and LCAP**

Study, first author	Year	Study type	Patients, n	Sex	Age (y)	Primary FSGS	biopsy	Previous treatment	Duration of treatment prior to EPT	EPT protocol	Concomitant treatment	Duration of follow-up	Responder, n/total (%) [a]	CR, n/total (%) [a]	PR, n/total (%) [a]	Outcomes [b]
Haas et al.	1998	Case series	5	3M 2F	1 child (16y girl); other 4 patients: 23, 25, 29, and 49y	yes	yes	NA	8-48 months	**IA** for 4 patients; **IgG-IA** for the 25y male; **5 sessions** within 10 days	NA	NA	2/5 (40)		2/5 (40)	• PR in 2/5 (**40%**; 49y female and 25y male; a reduction of proteinuria by >50% from 7 to 0.5 g/day and from 9 to 3 g/day, respectively)
Moriconi et al.	2001	Case series	7: 3 transplants; **4 native kidneys** (2 early FSGS, 1 late stage FSGS, 1 partially advanced FSGS)	2M 2F	range18-60y	yes	yes	All patients: CS (i.v: pulses and/or high oral daily dosage); 1 patient: CsA for 4 months; 1 patient: a brief cycle with AZA; 1 patient: a short period of CsA; 1 patient: Cy for 1 month	1-4 moths	IA; **10 sessions** in the intensive period of treatment within 4 weeks; no remission, stop IA; if remission, IA sessions frequency gradually reduced	Reduced IS or gradually discontinued	25 weeks in the patient with PR; 6 months in a patient with progression to ESKD; NA in other 2 patients	¼ (25) **(long-term)**		1/4 (25)	• PR in ¼ (**25%**): the patient with partially advanced FSGS had a clinical remission after a 25-weeks follow up period, showing normalization of permeability factor and SCr levels and a clear decrease in proteinuria (from 14 to 4 at week 8 and to 2 g/day at week 25)
Kuhn et al.	2006	Case reports	1	M	34	yes	yes	Failed on therapy with steroids, CP,	5 years (in the third year,	IA, **10 sessions** over a 4-week period (4 sessions	CP 750 mg/m^2	NA	1		1	• PR (a reduction of proteinuria from 9.2 to 4.5 g/day) and constant increase of serum albumin and reduction

Information Classification: General

								CsA, Tac and MMF	steroid bolus therapy plus a triple PE followed by AZA resulted in a protein reduction of 2,5 g/day)	within the first week followed by 2 sessions per week)						in serum cholesterol during IA<br>• 2 months after IA, relapsed with 19 g/day proteinuria. Continued IA 2 sessions a week for 4 weeks followed by once a week without IS, PR (a reduction of proteinuria by >50% from 19 to 3 g/day)
Yokoyama et al.	2002	Case series	6 (**2 FSGS**, 2 MCNS, 1 Mn, 1 MN and FSGS) (Japanese)	F	24 and 34	yes	yes	1 patient: no therapy, 1 patient: pulse/PSL and CsA	1 and 18 months	**LCAP**; twice in 2 consecutive weeks	The 24y old patient: no therapy during LCAP, but follow therapy with pulse/PSL and CsA for 47 months; The 34y old patient: a combination of LDL-A, PSL and CsA during LCAP and followe	47 and 40 months	½ (50) (**long-term**)	1		• Of the 2 FSGS, CR in 1/2 [c]<br>• Of the 2 MCNS, 2/2 CR<br>• 1 MN and FSGS: PR<br>• 1 MN: death by pneumonia

											d for 40 months					

Abbreviations: *EPT: extracorporeal plasma therapy; IA: immunoadsorption using protein A; IgG-IA: immunoadsorption using anti-IgG-antibodies; LCAP: lymphocytapheresis using Cellsorba, a leukapheresis filter; M: male; F: female; NA: not available; PE: plasmapheresis or plasma exchange using albumin; LDL-A: LDL apheresis using dextran sulfate cellulose column; CR: complete remission; PR: partial remission; SCr: serum creatinine; IS: immunosuppressive or immunosuppressants; CS: corticosteroids; CsA: cyclosporine; AZA: azathioprine; Tac: tacrolimus; RTX: rituximab; MMF: mycophenolate mofetil; PSL: prednisolone; Pulse: methylprednisolone pulse therapy*.

[a]The percentage of CR and PR is shown for case series studies.

[b] If not mentioned in the studies, CR and PR is determined by the alteration of proteinuria. CR is defined as reduction of proteinuria to protein excretion less than 0.3 g per 24 hours and PR as reduction of proteinuria to protein excretion of 0.3 to 3 g per 24 hours and 50% reduction in proteinuria from baseline.

[c] CR: daily proteinuria of less than 0.2 g with normal serum albumin levels; PR: normal serum albumin levels (more than 30 g/L) with a mean daily proteinuria of less than 3.5 g for 7 consecutive days.

References

1. Haas M, Godfrin Y, Oberbauer R, Yilmaz N, Borchhardt K, Regele H, et al. Plasma immunadsorption treatment in patients with primary focal and segmental glomerulosclerosis. Nephrol Dial Transplant. 1998;13(8):2013-6.
2. Moriconi L, Lenti C, Puccini R, Pasquariello A, Rindi P, Batini V, et al. Proteinuria in focal segmental glomerulosclerosis: role of circulating factors and therapeutic approach. Ren Fail. 2001;23(3-4):533-41.
3. Kuhn C, Kuhn A, Markau S, Kastner U, Osten B. Effect of immunoadsorption on refractory idiopathic focal and segmental glomerulosclerosis. J Clin Apher. 2006;21(4):266-70.
4. Yokoyama H, Shimizu M, Wada T, Yoshimoto K, Iwata Y, Shimizu K, et al. The beneficial effects of lymphocytapheresis for treatment of nephrotic syndrome. Ther Apher. 2002;6(2):167-73.

Information Classification: General
